# Arbeitsanforderungen und Ressourcen der digitalen Mediennutzung bei Lehrkräften: Eine Group-concept-mapping-Studie mit angehenden Lehrkräften

**DOI:** 10.1007/s11553-023-01015-w

**Published:** 2023-02-09

**Authors:** Malte Cramer, Ingmar Hosenfeld

**Affiliations:** Zentrum für Empirische Pädagogische Forschung, Rheinland-Pfälzische Technische Universität Kaiserslautern-Landau, Landau, Deutschland

**Keywords:** Lehrkräftegesundheit, Digitaler Stress, Wohlbefinden, Digitalisierung, Schule, Teacher health, Digital stress, Well-being, Digitalization, School

## Abstract

**Hintergrund:**

Die Nutzung digitaler Medien ist für einen Teil der Lehrkräfte mit einem erhöhten Belastungserleben verbunden. Stress, der durch negative Aspekte der digitalen Mediennutzung entsteht, wird unter dem Begriff „digitaler Stress“ oder „Technostress“ v. a. in internationalen Studien untersucht. Für deutsche Lehrkräfte ist die Befundlage hingegen rar. Dem gegenüberstehend scheint ein weiterer Teil der Lehrkräfte geringere Schwierigkeiten mit der fortschreitenden Implementierung digitaler Medien in den Schulalltag zu haben und stattdessen von den Vorteilen digitaler Medien zu profitieren. Empirisch vernachlässigt wurde dabei die Frage, inwiefern sich diese Vorteile als Ressource positiv auf Stress und Wohlbefinden der Lehrkräfte auswirken können.

**Ziel:**

Ziel der Studie ist es, eine Zusammenstellung von potenziellen Einflussfaktoren der digitalen Mediennutzung auf Stress und Wohlbefinden von Lehrkräften aus Deutschland zu erhalten, diese datengeleitet zu strukturieren und hinsichtlich ihrer Wichtigkeit zu bewerten.

**Methode:**

Es wurde die Group-concept-mapping(GCM)-Methode mit 44 angehenden Lehrkräften der Universität Koblenz-Landau durchgeführt.

**Schlussfolgerung:**

Die erzielte Strukturierung zeigt in Verbindung mit der bewerteten Wichtigkeit die Bedeutung positiver Auswirkungen der digitalen Mediennutzung für das Wohlbefinden von Lehrkräften sowie die Relevanz schulischer Rahmenbedingungen auf.

**Zusatzmaterial online:**

Zusätzliche Informationen sind in der Online-Version dieses Artikels (10.1007/s11553-023-01015-w) enthalten.

## Abstract (en)

**Background:**

The use of digital media is associated with an increased stress experience for some teachers. Stress caused by negative aspects of digital media use is investigated under the term *digital stress* or *technostress*, especially in international studies. Empirical evidence for German teachers, however, is scarce. In contrast, another group of teachers seem to have fewer difficulties with the progressive implementation of digital media in everyday school life and instead benefit from the advantages of digital media. Empirically, the question in how far these advantages can have a positive effect on teachers’ stress and well-being as a resource has been neglected.

**Aim:**

The aim of the study is to obtain a compilation of potential influencing factors of digital media use on stress and well-being of teachers, to structure them in a data-driven manner and to evaluate them with regard to their importance.

**Methods:**

The group concept mapping (GCM) method was conducted with 44 teacher trainees at the University of Koblenz-Landau, Germany.

**Conclusion:**

The achieved structuring in connection with the evaluated importance shows the relevance of positive effects of digital media use for teachers’ well-being as well as the relevance of school conditions.

## Einleitung

Die Digitalisierung an Schulen ist nicht erst seit der COVID-19(„coronavirus disease 2019“)-Pandemie ein Thema von höchster Aktualität. Spätestens seit dem Beschluss der Kultusministerkonferenz von 2016 und dem damit verknüpften DigitalPakt, der das politische Vorhaben finanziell unterstützen soll, wird sie vonseiten der Politik forciert und mit konkreten Zielvereinbarungen versehen [8, 29]. Auch wenn die Bilanz dieser Strategie zunächst ernüchternd ausfiel [26], kam es in den Pandemiejahren 2020/2021 zu einem regelrechten Digitalisierungsschub [34]. Insbesondere konnten neben einer häufigeren digitalen Mediennutzung der Ausbau der digitalen Infrastruktur, höhere digitale Kompetenzen der Lehrkräfte und eine fortschreitende Entwicklung digitaler Schulstrategien festgestellt werden, die konkrete Ziele sowie Wege zur Umsetzung der Digitalisierung festlegen [34].

Die Forschung zur Digitalisierung an Schulen beschränkte sich v. a. im deutschsprachigen Raum lange auf die Nutzungshäufigkeit digitaler Medien im Unterricht durch Lehrkräfte und auf Faktoren, die den Einsatz digitaler Medien im Schulkontext begünstigen [17, 42, 43]. Inwiefern sich die veränderte Arbeitsrealität auf die Lehrkräftegesundheit auswirkt, tritt erst seit Kurzem und speziell seit der COVID-19-Pandemie verstärkt in den Vordergrund. So konnte aufgezeigt werden, dass die Digitalisierung für einen Teil der Lehrkräfte mit einem hohen Stress und einer erhöhten Arbeitsbelastung verbunden ist [32, 34]. Als Ursachen werden in diesen Studien u. a. eine ohnehin schon hohe Arbeitsintensität genannt, die durch die Digitalisierung noch einmal potenziert würde, sowie eine erschwerte Trennung von Arbeit und Freizeit aufgrund der ständigen Erreichbarkeit über digitale Kommunikationsmittel. Es werden jedoch auch positive Aspekte und Potenziale digitaler Medien im wissenschaftlichen Diskurs angeführt, die Stress reduzieren und Wohlbefinden fördern könnten, wie eine Zeitersparnis oder eine Nützlichkeit für das Lernen der Schüler*innen [10, 32]. Zusammengenommen ist die Befundlage v. a. zu positiven Folgen jedoch spärlich [37].

Bei Untersuchungen zur Lehrkräftegesundheit ist zu beachten, dass die beruflichen Bedingungen und die Ausbildung der Lehrkräfte zwischen Ländern stark variieren können [28]. Die vorliegende Studie möchte daher die Befundlage für angehende Lehrkräfte in Deutschland erweitern. Über die Verbindung von qualitativen und quantitativen Methodenelementen sollen durch die Group-concept-mapping(GCM)-Methode nach Trochim [50] Arbeitsanforderungen und Ressourcen der digitalen Mediennutzung bei Lehrkräften durch angehende Lehrkräfte identifiziert und strukturiert werden. Ziel ist es weiterhin, die Faktoren hinsichtlich ihrer Wichtigkeit zu beurteilen und mit dem aktuellen Forschungsstand in Bezug zu setzen.

## Theorie und Forschungsstand

### Stress und Wohlbefinden bei der Arbeit

Nach den Ergebnissen der aktuellen Stressstudie der Techniker Krankenkasse [48] nimmt der Stress in Deutschland stetig zu. Einer der häufigsten Gründe stellt dabei die Arbeitssituation dar. Dies ist problematisch, da Menschen viel Zeit ihres Lebens am Arbeitsplatz verbringen und negative Auswirkungen von Stress auf die psychische und physische Gesundheit bekannt sind [7, 40]. Wohlbefinden umfasst nach der Definition der Weltgesundheitsorganisation (WHO) „die Gesamtheit menschlicher Lebensbereiche, einschließlich physischer, mentaler und sozialer Aspekte […]“ [13, S. 144]. Damit ist auch die Arbeitssituation für das Wohlbefinden von Relevanz, das wiederum – wie auch Stress – für die Gesundheit des Individuums von Bedeutung ist [13]. Wie es zu Stress und Wohlbefinden im Zuge der Arbeit kommt, kann mit Hilfe des *Arbeitsanforderungen-Arbeitsressourcen-Modells* [12] dargelegt werden. In diesem Modell werden negative Faktoren der Arbeit, die zu Stress und langfristig zu Burnout führen, unter dem Begriff Arbeitsanforderungen zusammengefasst. Hierunter fallen z. B. Konflikte am Arbeitsplatz oder Schichtarbeit. Positive Faktoren hingegen, wie beispielsweise ein unterstützendes Arbeitsumfeld oder vorhandene Handlungsspielräume, fördern das Arbeitsengagement und werden als Arbeitsressourcen bezeichnet. Diese Ressourcen stehen wiederum in einem positiven Rückkopplungsprozess mit Wohlbefinden [12]. Arbeitsanforderungen und -ressourcen stehen in Wechselwirkung zueinander, sodass die vorhandenen Ressourcen den negativen Einfluss von Arbeitsanforderungen auf Stress verringern können. Die ursprünglich ausschließlich auf die Arbeit bezogenen Ressourcen wurden nachträglich um personale Ressourcen erweitert. Damit sind nicht allein die Merkmale der Tätigkeit für die Frage wichtig, welche Arbeitsanforderungen und -ressourcen in einem spezifischen Arbeitskontext wirksam sind, sondern auch die Eigenschaften des einzelnen Individuums, wie beispielsweise dessen Selbstwirksamkeit [12].

### Digitaler Stress als spezifische Stressart

Stress, der durch negative Folgen der digitalen Mediennutzung entsteht, wird unter den Begriffen „technostress“ [39], „digital stress“ [20] oder in deutschen Veröffentlichungen „digitaler Stress“ [16, 34] diskutiert. Technostress wurde dabei ursprünglich von Brod als „a modern disease of adaptation caused by an inability to cope with the new computer technologies in a healthy manner“ [6, S. 16] definiert. Aktuellere Definitionen verstehen unter dem Begriff spezifische Belastungen, die durch die Nutzung digitaler Medien entstehen [14, 45]. So müssen sich Arbeitende beispielsweise zunächst die Kompetenzen aneignen, um neue digitale Medien bedienen und in die Arbeit integrieren zu können, was zu einer erhöhten Arbeitsintensität führen kann. Weiterhin kann die Digitalisierung einen Einfluss darauf nehmen, wie (zusammen)gearbeitet wird – Veränderungen, die Arbeitende als negativ auffassen können [46]. Fest steht, dass digitaler Stress durch die fortschreitende Digitalisierung zu einem Forschungsthema von hoher Relevanz in verschiedenen Arbeitskontexten geworden ist [35]. Empirische Studien bringen dabei digitalen Stress mit Burnout und psychischer Erschöpfung [9, 22, 36], einem schlechteren allgemeinen Gesundheitszustand [22], geringerer Arbeitszufriedenheit [22, 24, 47], geringerer beruflicher Leistung [2, 22, 47] und Kündigungsabsichten [47] in Zusammenhang.

### Digitaler Stress bei Lehrkräften

Auch Lehrkräfte sind von der fortschreitenden Digitalisierung betroffen. Sie müssen Schülerinnen und Schülern früh digitale Kompetenzen vermitteln, die als eine zentrale Voraussetzung für einen erfolgreichen Bildungsweg und für die Partizipation in der Gesellschaft gesehen werden [29]. Da dies fächerübergreifend erfolgen soll, betrifft dies das gesamte Kollegium. Konkrete digitale Kenntnisse, Fähigkeiten und Kompetenzen, die Schülerinnen und Schüler nach ihrer Pflichtschulzeit besitzen sollen, wurden in einem Beschluss der Kultusministerkonferenz aus dem Jahr 2016 definiert und in 6 Kompetenzbereiche unterteilt [29]. Diese behandeln nicht nur das einfache Recherchieren von digitalen Informationen, sondern auch komplexere Fähigkeiten wie das Produzieren digitaler Produkte unter Berücksichtigung rechtlicher Vorgaben. Wie Mußmann et al. [34] anmerken, stehen Lehrkräfte damit vor einer doppelten Herausforderung: Zum einen sollen digitale Medien didaktisch sinnvoll eingesetzt und Schüler*innen auf eine digitale (Arbeits)welt vorbereitet werden. Zum anderen wird erwartet, dass Lehrkräfte sich die entsprechenden Kompetenzen zur Nutzung und Integration digitaler Medien aneignen und sich fortlaufend fortbilden. Dabei hängt der Erfolg, mit der Situation umzugehen, nicht allein von der einzelnen Lehrkraft ab. Externe Faktoren wie bildungspolitische Vorgaben und schulische Rahmenbedingungen können die individuellen Gestaltungsmöglichkeiten des Digitalisierungsprozesses einschränken [52].

Zu den gesundheitlichen Folgen der Digitalisierung existieren für Lehrkräfte aus Deutschland 2 Studien. In einer Befragung von Mauss [32] gab mehr als ein Drittel der Lehrkräfte an, durch digitale Medien eine höhere unterrichtliche Arbeitsbelastung zu erfahren. Bei Mußmann et al. [34] gaben Lehrkräfte im Vergleich zu anderen Berufsgruppen höhere digitale Stresswerte an. Über alle Facetten des digitalen Stresses hinweg lagen die gemessenen Werte im mittleren Bereich. Die Autoren betonen jedoch, dass ihre Daten während der COVID-19-Pandemie erhoben wurden, die Daten der Vergleichsstudien jedoch nicht. Internationale Studien, die vor der COVID-19-Pandemie durchgeführt wurden, zeigen jedoch ebenfalls ein mittleres Level an digitalem Stress bei Lehrkräften an [11, 35, 45].

### Welche Einflussfaktoren können bei der digitalen Mediennutzung von Lehrkräften wirksam sein?

Aus Deutschland liegen zwei Untersuchungen vor, die der Frage nachgehen, welche Faktoren im Zuge der digitalen Mediennutzung von Lehrkräften als belastend erlebt werden. In der bereits genannten Befragung von Mauss [32] gab ein Teil der Lehrkräfte an, dass die Nutzung digitaler Kommunikationsmittel außerhalb von Unterrichtszeiten sowie eine erschwerte Trennung von Arbeit und Freizeit Arbeitsanforderungen darstellen würden. Zudem spielte die Nutzenwahrnehmung eine wichtige Rolle: Lehrkräfte, die digitale Medien als nützlich für den Unterricht empfanden, berichteten über eine geringere Arbeitsbelastung. Ausführlicher wurden verschiedene Einflussfaktoren bei Mußmann et al. [34] untersucht. Hier gingen die digitalen Kompetenzen und die IT-Affinität der Lehrkräfte, d. h. inwiefern sich diese aus eigener Motivation heraus mit (neuen) digitalen Medien beschäftigen und digitale Medien in ihre Lehre integrieren, mit niedrigerem digitalen Stress einher. Von Relevanz ist zudem die schulische Unterstützung. Hier erreichten Lehrkräfte signifikant höhere Stresswerte, wenn die schulische Unterstützung bezüglich der Implementierung digitaler Medien als nicht ausreichend empfunden wurde. Weiterhin kategorisierten die Autoren Schulen hinsichtlich der Entwicklung einer digitalen Schulstrategie und des Aufbaus einer digitalen Infrastruktur. Lehrkräfte aus Schulen mit einer weiter entwickelten digitalen Schulstrategie und einer besseren digitalen Infrastruktur erzielten dabei unter anderem signifikant geringere digitale Stresswerte. Schließlich stand digitaler Stress mit einer erhöhten Beanspruchung aus der Arbeitssituation (u. a. gekennzeichnet durch eine hohe Arbeitsintensität und geringe arbeitsbezogene Handlungsspielräume) in Zusammenhang. Die Autoren schließen daraus, dass Lehrkräfte oftmals aufgrund der schulischen Gegebenheiten nicht die Möglichkeiten haben, sich angemessen mit Digitalisierungsprozessen zu beschäftigen, was digitalen Stress zur Folge haben kann.

Ein breiteres Bild ergibt sich, wenn internationale Studien hinzugezogen werden. Hier ist der am häufigsten genannte Einflussfaktor die digitale Kompetenz der Lehrkraft [1, 14, 25, 35, 45]. Die genannten Studien zeigen kongruent mit den Befunden von Mußmann et al. [34] einen negativen Zusammenhang des Konstrukts mit digitalem Stress auf. Weitere Studien berichten negative Zusammenhänge zwischen digitalem Stress und schulischer Unterstützung [25, 35], positiver Einstellung gegenüber digitalen Medien [45], (computerbezogener) Selbstwirksamkeit [14, 19], Übereinstimmung des pädagogischen Einsatzes digitaler Medien mit dem Lehrstil [45], Häufigkeit der Internetnutzung [1, 11], technische Ausstattung und Integrierbarkeit von digitalen Medien in den Unterricht [31]. Positive Zusammenhänge konnten zwischen digitalem Stress und Mehrarbeit und Computerangst [18] sowie Zeitaufwand für die Vorbereitung von digitalem Unterricht [31] aufgezeigt werden. Zu der demographischen Variable Alter zeigen einzelne internationale Befunde, dass Lehrkräfte höheren Alters höhere digitale Stresswerte aufweisen [35, 45]. Bezüglich des Geschlechts berichteten weibliche Lehrkräfte bei Syvänen et al. [45] mehr digitalen Stress, wohingegen in anderen Studien keine geschlechtsspezifischen Unterschiede gefunden wurden [11, 35]. In der Studie von Al-Fudail [1] konnten Lehrkräfte zudem offene Antworten zu Einflussfaktoren von digitalem Stress geben. Im Einklang mit den Ergebnissen der quantitativen Befragungen wurden am häufigsten die fehlenden digitalen Kompetenzen der Lehrkräfte als Stressor angeführt, gefolgt von fehlender Unterstützung, technischen Problemen und Zeitverschwendung durch die Verwendung schlechter Software.

Zusammenfassend kann festgestellt werden, dass v. a. internationale Studien verschiedene Einflussfaktoren zu digitalem Stress bei Lehrkräften empirisch untersucht haben. Neben Faktoren, die sich wie die digitalen Kompetenzen auf die einzelne Lehrkraft beziehen, werden die schulischen Voraussetzungen bei der Entstehung von digitalem Stress hervorgehoben.

### Digitale Mediennutzung als Entlastung für Lehrkräfte?

Verschiedene Studien, die sich nicht konkret auf Lehrkräfte beziehen, konnten positive Effekte digitaler Medien auf die Gesundheit von Arbeitenden aufzeigen. Bei Hardwig [23] ging beispielsweise eine intensivere Nutzung von digitalen Kollaborationsplattformen mit einer höheren Arbeitszufriedenheit und günstigeren psychischen Gesundheit einher. In einer weiteren Studie wurde eine erhöhte Arbeitsflexibilität durch digitale Medien mit einem höheren Wohlbefinden in Zusammenhang gebracht, das über eine bessere Work-Life-Balance, größere Autonomie und effektivere Kommunikation vermittelt wurde [49]. In Studien zur Lehrkräftegesundheit wurden positive Folgen der digitalen Mediennutzung hingegen bisher vernachlässigt [37]. Empirisch wurde die Auswirkung digitaler Kommunikation auf die Eltern-Lehrkraft-Beziehung untersucht [30]. Den Ergebnissen zufolge kann die Beziehung durch digitale Kommunikation – insbesondere in ländlichen Gebieten – verbessert werden, was sich wiederum positiv auf das Wohlbefinden von Lehrkräften auswirken könnte. In einer weiteren Studie wurde u. a. der Zusammenhang von Persönlichkeitseigenschaften (Extraversion, Gewissenhaftigkeit und Neurotizismus), digitalen Kompetenzen und dem Wohlbefinden von Lehrkräften regressionsanalytisch untersucht [44]. Die digitalen Kompetenzen stellen demnach über die Persönlichkeitseigenschaften hinaus einen signifikanten Prädiktor dar, auch wenn der Anteil zusätzlich aufgeklärter Varianz gering war. Die Autorin nimmt an, dass Lehrkräfte mit hohen digitalen Kompetenzen zuversichtlicher und weniger gestresst bei der Nutzung digitaler Medien sind, was zu einem besseren berufsbezogenen Wohlbefinden und geringerem Burnout führen könnte. Darüber hinaus existieren Befunde, die Vorteile der digitalen Mediennutzung für Lehrkräfte ohne direkten Bezug zu Wohlbefinden aufzeigen. Diese könnten ebenfalls relevant sein, um Ressourcen der digitalen Mediennutzung für Lehrkräfte zu identifizieren. In der Studie von Mauss [32] empfand eine Mehrheit der Lehrkräfte digitale Medien für die Unterrichtsgestaltung und das Lernen immer oder häufig nützlich, während bei Mußmann et al. [34] eine bessere Arbeitseffizienz und eine Professionalisierung des Unterrichts berichtet wurde, da beispielsweise besser auf die Bedürfnisse einzelner Schüler*innen eingegangen werden kann. In weiteren Studien wurde eine Zeitersparnis durch digitale Medien in der Schule hervorgehoben [10, 32, 52]. Beispielsweise wurde die Möglichkeit, Unterrichtsmaterialien auf einer Online-Plattform ablegen zu können, von Lehrkräften als zeitsparend empfunden [10]. Schulleitungen berichten zudem von einer Vereinfachung von verwaltungstechnischen Aufgaben durch digitale Medien [52]. Die Befunde sind jedoch nicht eindeutig: Bei Mauss [32] waren Lehrkräfte in der Frage des Zeitaufwands für die Unterrichtsgestaltung gespalten. Ungefähr ein Drittel gab jeweils an, dass digitale Medien eher Zeit kosten, eher Zeit sparen bzw. keinen Unterschied machen würden. Schließlich werden eine bessere Zusammenarbeit über Netzwerke mit anderen Lehrkräften [52], Unterstützung konstruktivistischen Lernens (z. B. durch selbstständiges Lernen und Übernahme eigener Verantwortung der Schüler*innen), Entlastung der Lehrkraft von Lehrfunktionen (z. B. durch automatische Korrekturfunktionen) und Förderung der Lernmotivation von Schüler*innen [43] als Vorteile digitaler Medien genannt.

Insgesamt kann festgestellt werden, dass kaum empirische Befunde zu den wahrgenommenen Effekten der Vorteile digitaler Medien auf das Wohlbefinden von Lehrkräften vorliegen. Im Gegensatz dazu wird eine Vielzahl an Vorteilen digitaler Medien hervorgehoben, die als Ressource das Wohlbefinden von Lehrkräften fördern könnten.

### Ziele und Forschungsfragen

In Hinblick auf den vorgestellten Forschungsstand zu be- und entlastenden Faktoren der digitalen Mediennutzung bei Lehrkräften möchte die vorliegende Studie die Befundlage für deutsche Lehrkräfte erweitern. Hierfür sollen angehende Lehrkräfte Faktoren generieren, die bei der Nutzung digitaler Medien im schulischen Kontext im Zusammenhang mit der Befindlichkeit von Lehrkräften stehen könnten. Der Begriff Befindlichkeit wurde gewählt, damit sowohl Arbeitsanforderungen als auch Ressourcen von den Studienteilnehmenden berücksichtigt und untersucht werden können. Diese sollen in einem nächsten Schritt datengeleitet strukturiert werden, um einen Überblick über diese Einflussfaktoren bereitzustellen. Schließlich sollen die Faktoren hinsichtlich ihrer Wichtigkeit bewertet werden, um ein Kriterium zur Differenzierung von relevanten bzw. weniger relevanten Faktoren zu erhalten. Zusammenfassend sollen explorativ die folgenden Fragestellungen beantwortet werden:

1. Welche Arbeitsanforderungen und Ressourcen könnten bei der Nutzung digitaler Medien im schulischen Kontext von Lehrkräften relevant sein?
2. Wie lassen sich diese Faktoren strukturieren?
3. Welche Faktoren werden als besonders wichtig oder unwichtig eingeschätzt?

## Methode

Zur Beantwortung der Forschungsfragen wird die GCM-Methode nach Trochim [50] mit angehenden Lehrkräften angewendet. Da die Methode insbesondere im deutschsprachigen Raum nicht weit verbreitet ist, folgt eine kurze Darstellung des Durchführungsprozesses.

### „Group concept mapping“

**Abb. 1:**
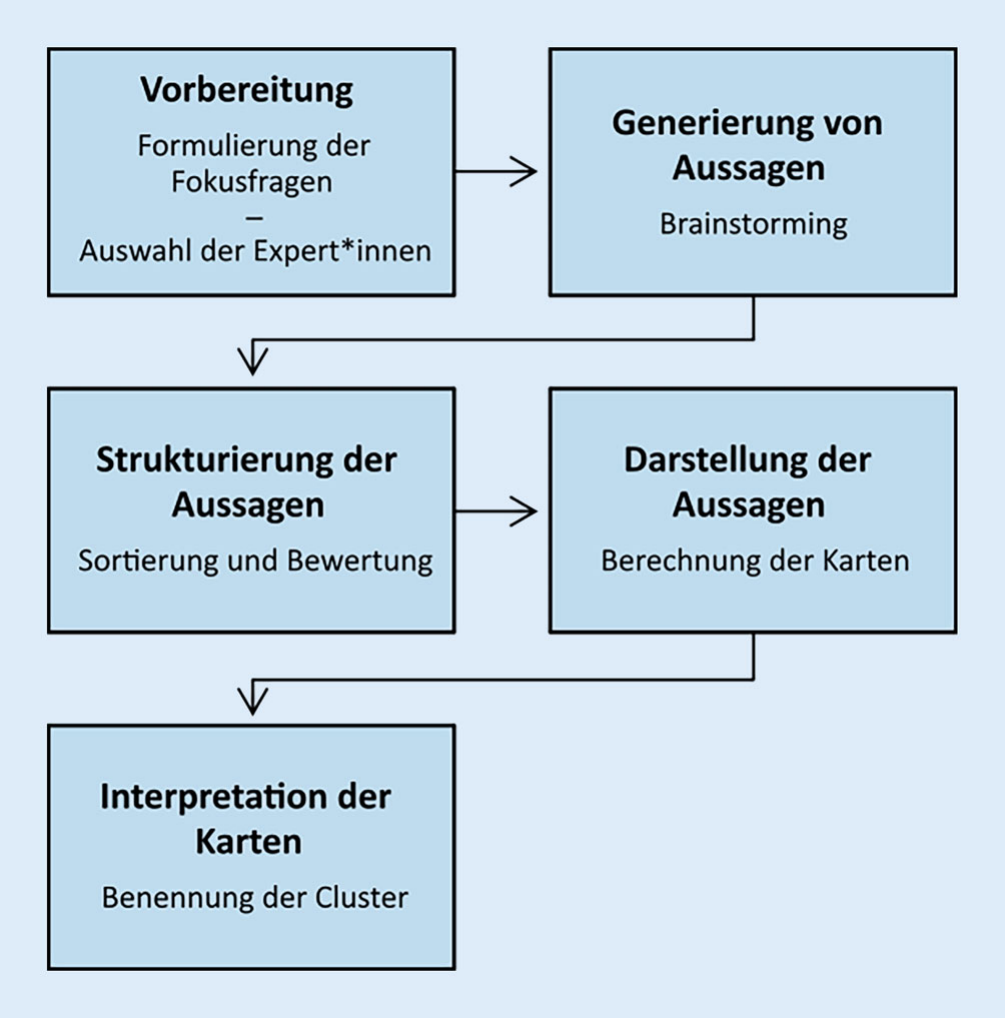
Phasen des „group concept mapping“ (GCM)

Bei dem GCM werden qualitative und quantitative Methodenelemente kombiniert [50]. Eine Teilnehmergruppe, die in Bezug auf das Erkenntnisinteresse der Studie Expertenwissen aufweist, generiert Aussagen und Ideen zu einer vorher definierten Fokusfrage. Diese werden von der Studienleitung und einer Teilgruppe der Expert*innen auf redundante und eindeutig nicht relevante Aussagen geprüft und gegebenenfalls eliminiert. Nachdem die Teilnehmenden im Anschluss den finalen Aussagensatz erhalten haben, werden die Aussagen von den Teilnehmenden auf Stapel sortieren. Die Sortierungslogik wird nicht vorgeschrieben, vielmehr sollen die Teilnehmenden die Aussagen so sortieren, wie es ihnen sinnvoll erscheint. Es gelten lediglich folgende Einschränkungen: 1. Jede Aussage darf nur auf einen Stapel gelegt werden. 2. Alle Aussagen dürfen nicht auf denselben Stapel gelegt werden. 3. Nicht alle Aussagen dürfen alleine auf einem Stapel gelegt werden. Zudem werden die Aussagen von den Teilnehmenden nach zuvor definierten Kriterien bewertet. Über nichtmetrische Multidimensionale Skalierung wird aus den einzelnen Sortierungen eine zweidimensionale kartenartige Repräsentation erstellt, die „concept map“ (CM). Auf dieser werden Aussagen als Punkte dargestellt, wobei Aussagen, die häufiger zusammen sortiert wurden, näher beieinander liegen. Durch clusteranalytische Verfahren können im Anschluss Gruppen von Aussagen zusammengefasst werden, um so die CM einfacher interpretieren zu können. Nach der Generierung interpretieren und diskutieren die Teilnehmenden die erzeugte CM. Jedes Cluster soll von den Teilnehmenden mit einem Schlagwort oder kurzem Satz benannt werden, der den Inhalt ihrer Ansicht nach gut beschreibt. Es wird versucht, im Rahmen einer Gruppendiskussion einen Konsens über die Benennung der Cluster zu finden. Zur weiteren Analyse der Karte können die Dimensionen der CM interpretiert werden [5, 27]. Abb. 1 gibt einen Überblick über die einzelnen Phasen des Prozesses.

### Stichprobenbeschreibung

Die akquirierte Gesamtstichprobe umfasste 44 angehende Lehrkräfte, von denen 75 % weiblichen und 25 % männlichen Geschlechts waren. Der Altersdurchschnitt lag bei 21,77 Jahren (*SD* = 1,92). 82 % der Teilnehmenden gaben an, über das erste orientierende Praktikum hinaus weitere praktische Unterrichtserfahrung (z. B. als Vertretungslehrkraft) zu haben. Als Vergütung wurden Leistungspunkte für das Studium vergeben.

### Durchführungsprozess

Die Fokusfrage für die Aussagengenerierung lautete: „Beschreiben Sie in Stichworten, welche Faktoren bei der Nutzung digitaler Medien im schulischen Kontext im Zusammenhang mit der Befindlichkeit von Lehrkräften stehen könnten“. Da der Begriff Befindlichkeit sowohl positive als auch negative Gefühle umfassen kann, wurde dieser verwendet, um die Expert*innen anzuregen, sowohl positive als auch negative Faktoren zu erzeugen. Die Aufgabe für die Bewertung der einzelnen Aussagen lautete: „Bewerten Sie bitte jede generierte Aussage auf einer 4‑stufigen Skala (von 1 = *unwichtig *bis 4 = *wichtig*) dahingehend, wie wichtig Sie diese für den Zusammenhang zwischen der Nutzung digitaler Medien im Schulkontext und der Befindlichkeit von Lehrkräften halten“.

Als Expert*innen wurden im Mai 2021 über den E‑Mail-Verteiler der Universität Koblenz-Landau angehende Lehrkräfte für allgemeinbildende Schulen angeworben, die mindestens das erste orientierende Praktikum abgeschlossen hatten. Dieses Kriterium sollte gewährleisten, dass die Teilnehmenden praktische Erfahrung als Lehrkraft besitzen.

In einer Web-Videokonferenz über BigBlueButton [4] wurden allen Teilnehmenden die zentralen Begrifflichkeiten der Aufgabenstellung erläutert. Die Generierung der Aussagen erfolgte im Anschluss an die Sitzung in Einzelarbeit über die virtuelle Plattform ONCOO („Online Cooperation“) [33]. Es erzeugten 44 teilnehmende Expert*innen 228 verschiedene Aussagen. Diese wurden von der Studienleitung auf inhaltliche Doppelungen sowie auf ihre Relevanz hin überprüft, sodass 133 Aussagen übrigblieben. Da Aussagen erzeugt wurden, deren Bedeutung vom Studienleiter nicht eingeschätzt werden konnte, wurde zudem mit einer Teilgruppe von Studienteilnehmenden (*n* = 12) eine weitere Videokonferenz durchgeführt. Jede*r Teilnehmende erhielt hierfür im Vorhinein einen Link zu ONCOO, auf der die Aussagen bereits vor der Videokonferenz eingesehen und markiert werden konnten, wenn sie den Teilnehmenden als diskussionswürdig erschienen. Es wurden 41 von 133 Aussagen von mindestens einem Teilnehmenden als problematisch angesehen. Weitere 27 Aussagen wurden von dem Studienleiter zur Diskussion gestellt. Im Rahmen der Sitzung wurden schließlich acht Aussagen umbenannt und 33 entfernt.

Die Sortierung der Aussagen erfolgte ebenfalls über ONCOO. Zusätzlich sollten die Teilnehmenden die Aussagen ihrer Wichtigkeit nach einschätzen. Von 44 Teilnehmenden schickten 38 Teilnehmende dem Studienleiter ihre Ergebnisse zu. Unvollständige und fehlerhafte Datensätze wurden nicht berücksichtigt, sodass für die Sortierung 30 Datensätze und für die Bewertung der Aussagen 36 Datensätze berücksichtigt wurden.

Die Erstellung der Konzeptkarten über nichtmetrische Multidimensionale Skalierung und hierarchischer Clusteranalyse wurde mit dem Programm R‑CMap realisiert, das in R implementiert ist [3, 38]. Wie bei Kane und Trochim [27] vorgeschlagen, wurden Clusterlösungen iterativ absteigend hinsichtlich ihrer Interpretierbarkeit untersucht. Zudem wurde das Single-linkage-Verfahren der Clusteranalyse angewendet, um Aussagen zu identifizieren, die sich nur schlecht in die Karte integrieren ließen [53]. Eine Aussage wurde daraufhin entfernt, da diese inhaltlich keinem naheliegenden Cluster sinnvoll zugeordnet werden konnte. Für die einzelnen Aussagen und Cluster wurde schließlich die durchschnittlich eingeschätzte Wichtigkeit über R [38] berechnet.

Die Cluster wurden von einer weiteren Teilgruppe von Teilnehmenden (*n* = 10) in einer weiteren Videokonferenz diskutiert und mit Namen versehen. Die Studienleitung prüfte im Anschluss an die Diskussion die Passung der einzelnen Aussagen zu den benannten Clustern. Da eine Aussage als abweichend klassifiziert wurde, wurde diese nachträglich aufgrund einer besseren Passung von dem Cluster 4 in das Cluster 3 hinzugefügt.

## Ergebnisse

**Abb. 2:**
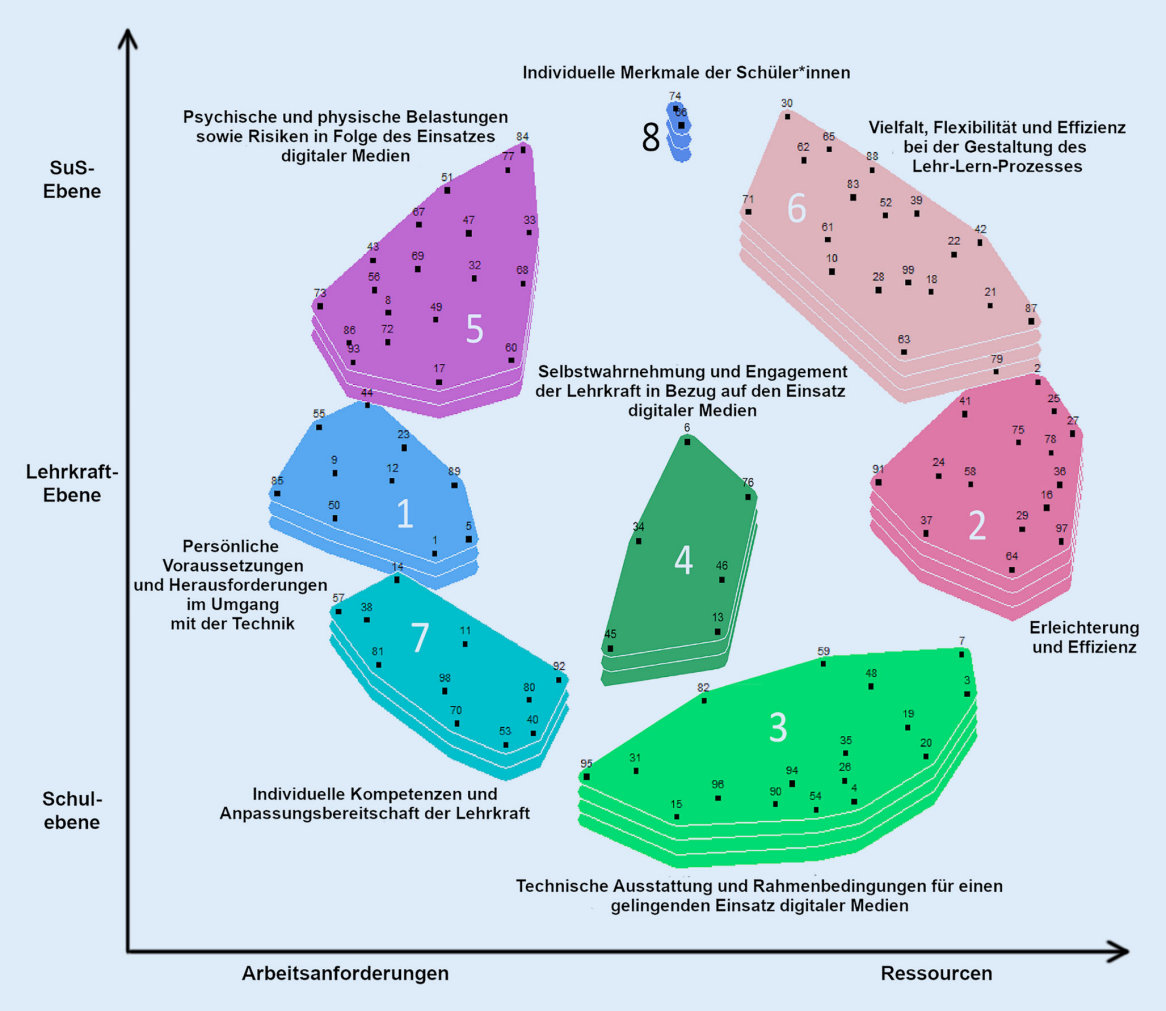
„Concept map“ (CM) mit 8 Clustern und den interpretierten Dimensionen. Die Höhe der Cluster zeigt Unterschiede in der bewerteten Wichtigkeit an. *SuS* Schülerinnen und Schüler

Unter Berücksichtigung der inhaltlichen Interpretierbarkeit der Cluster erschien eine Clusterlösung bestehend aus acht Clustern am besten geeignet. Der berechnete Fehleranteil, der angibt, wie gut die Abstände der generierten Karte die Sortierungen abbilden, beträgt 0,309 [5]. Ein niedriger Wert spiegelt eine gute Passung zwischen den Sortierungen und der erzeugten Karte wider. Damit liegt der Fehleranteil im Rahmen der berichteten durchschnittlichen Werten in anderen GCM-Projekten [15, 41, 51]. Im Folgenden werden der Inhalt der Cluster und einzelne Markeritems vorgestellt, die für die Cluster repräsentativ sind^1^. Zudem wird die Verortung des Clusters auf der bipolaren Bewertungsskala berichtet, die sich aus der bewerteten Wichtigkeit aller zugehörigen Aussagen ergibt. Schließlich werden die Dimensionen der CM interpretiert. Abb. 2 zeigt die finale CM.

Cluster 1 hat von den Teilnehmenden die Bezeichnung „Persönliche Voraussetzungen und Herausforderungen im Umgang mit der Technik“ erhalten. Konkret beziehen sich diese zum einen auf (problematische) persönliche Voraussetzungen, die für eine erfolgreiche Anwendung digitaler Medien hinderlich sind (A50^2^: „Technikangst“). Zum anderen werden Herausforderungen genannt, die insbesondere auf fehlende digitale Kompetenzen von Lehrkräften abzielen (A1: „Lehrkraft muss immer auf dem neusten Stand sein und sich immer weiter fortbilden“). Der Mittelwert der bewerteten Wichtigkeit aller Aussagen des Clusters liegt bei 2,52 (*SD* = 0,53) und entspricht damit ungefähr dem Neutralpunkt der bipolaren Skala. Dementsprechend empfanden die Teilnehmenden im Durchschnitt den Cluster als weder unwichtig noch wichtig.

Cluster 2 wurde die relativ allgemeine Bezeichnung „Erleichterung und Effizienz“ zugewiesen. Die enthaltenen Aussagen beschreiben Vorteile digitaler Medien für verwaltungs- und organisationsbezogene Aufgaben. Insbesondere werden eine Zeitersparnis für die Vor- und Nachbereitung des Unterrichts (A27: „Materialaustausch über Online-Plattformen erspart Zeit“) sowie einfache Kommunikationsmöglichkeiten (A16: „Erleichterung der Kommunikation mit Erziehungsberechtigten und Kolleg*innen“) thematisiert. Mit einem Mittelwert von 2,77 (*SD* = 0,38) tendiert der Cluster insgesamt zum Pol *wichtig* auf der Bewertungsskala.

Cluster 3 hat die Bezeichnung „Technische Ausstattung und Rahmenbedingungen für einen gelingenden Einsatz digitaler Medien“ erhalten. In diesem werden neben der technischen Ausstattung der Schule und einer funktionierenden digitalen Infrastruktur (A54: „W-LAN Empfang in der Schule“) unter anderem die vorhandenen Unterstützungsangebote bei digitalen Fragen (A7: „Hilfsangebote im Kollegium“) und Fortbildungsmöglichkeiten (A96: „Angebot schulischer Fortbildungen“) angesprochen. Insgesamt ist der Cluster mit einem Mittelwert von 3,12 (*SD* = 0,40) auf der Bewertungsskala in Richtung des Pols *wichtig* verortet.

Cluster 4 wurde mit „Selbstwahrnehmung und Engagement der Lehrkraft in Bezug auf den Einsatz digitaler Medien im Unterricht“ betitelt. Er besteht zum großen Teil aus Aussagen, die auf Eigenschaften der einzelnen Lehrkraft abzielen, die eine erfolgreiche Implementierung digitaler Medien unterstützen (A6: „Motivation, neue Dinge auszuprobieren“). Mit einem Mittelwert von 2,58 (*SD* = 0,19) tendiert das Cluster zum Neutralpunkt der bipolaren Skala.

Cluster 5 erhielt die Bezeichnung „Psychische und physische Belastungen sowie Risiken in Folge des Einsatzes digitaler Medien“. Im Gegensatz zum benachbarten Cluster 1, in dem insbesondere für die digitale Mediennutzung hinderliche Voraussetzungen beschrieben werden, beinhaltet dieser Cluster vor allem konkrete (negative) Auswirkungen der digitalen Mediennutzung auf die psychische und physische Gesundheit der Lehrkräfte (A84: „Augenschmerzen und Kopfschmerzen durch hohe Bildschirmzeit“). Mit einem Mittelwert von 2,50 (*SD* = 0,35) liegt das Cluster genau auf dem Neutralpunkt der bipolaren Bewertungsskala.

Cluster 6 hat die Überschrift „Vielfalt, Flexibilität und Effizienz bei der Gestaltung des Lehr-Lern-Prozesses“ erhalten. Der Cluster ähnelt inhaltlich dem Cluster 2, thematisiert jedoch Vorteile digitaler Medien für das Lehren und Lernen im Rahmen des Unterrichts (A18: „vielfältige Möglichkeiten der Unterrichtsgestaltung“) statt verwaltungs- und organisationsbezogene Aspekte im außerunterrichtlichen Kontext. Der Mittelwert des Clusters liegt bei 2,98 (*SD* *=* 0,24), sodass er in Richtung des Skalenpols *wichtig* verortet ist.

Cluster 7 wurde der Titel „Individuelle Kompetenzen und Anpassungsbereitschaft der Lehrkraft“ zugewiesen. Die Aussagen in dem Cluster thematisieren die digitalen Kompetenzen der Lehrkraft (A40: „Wissen von Anwendungsmöglichkeiten“) sowie Faktoren, die auf die Anpassungsbereitschaft der Lehrkraft im Zuge des digitalen Wandels abzielen (A81: „Zufriedenheit mit traditionellem Unterricht“). Damit ähnelt der Cluster dem benachbarten Cluster 1, in dem für eine erfolgreiche Implementierung digitaler Medien hinderliche individuelle Voraussetzungen und Herausforderungen genannt werden. Im Gegensatz zu diesem beinhaltet Cluster 7 jedoch nur wenige Aussagen, die sich auf hinderliche Faktoren beziehen, sondern stellt vielmehr eine Auflistung von relativ allgemeinen Faktoren dar (z. B. A92: „allgemeines Interesse am Umgang mit digitalen Medien“), die für die Integration digitaler Medien in die Schule relevant sind. Insgesamt erzielt der Cluster einen Mittelwert von 2,71 (*SD* = 0,49) auf der Bewertungsskala und neigt damit zum Pol *wichtig*.

Cluster 8 erhielt die Bezeichnung „Individuelle Merkmale der Schüler*innen“. Dieser Cluster besteht lediglich aus zwei Aussagen, die sich auf Eigenschaften der Schüler*innen beziehen (A66: „computerbezogenen Kompetenzen von Schüler*innen“; A74: „Alter der Schüler*innen“). Mit einem Mittelwert von 2,83 (*SD* = 0,94) ist der Cluster in Richtung des Pols *wichtig *verortet.

Wie zuvor erläutert, können darüber hinaus Dimensionen auf der CM interpretiert werden, die den Raum aufspannen und weitere Informationen zur Anordnung der CM liefern. Eine Dimension, die entlang der x‑Achse verläuft, kann danach interpretiert werden, ob die Aussagen eher Arbeitsanforderungen (linker Pol) oder Ressourcen (rechter Pol) im Zuge der digitalen Mediennutzung bei Lehrkräften beschreiben. Eine zweite mögliche Dimension auf der y‑Achse ordnet die Faktoren danach ein, ob diese eher von der Ebene der Schüler*innen, der Lehrkraft oder der Schule her wirken.

## Diskussion

Ziel der Studie war es, potenzielle Arbeitsanforderungen und Ressourcen der digitalen Mediennutzung im schulischen Kontext bei Lehrkräften zu identifizieren, zu strukturieren und hinsichtlich ihrer Wichtigkeit zu bewerten. Dazu erzeugten die Teilnehmer*innen eine Vielzahl an Aussagen, die ihrer Ähnlichkeit nach in Cluster zusammengefasst werden konnten.

Betrachtet man die CM in Hinblick auf die interpretierten Dimensionen, so lässt sich feststellen, dass das Verhältnis von Aussagen zu Arbeitsanforderungen und Ressourcen relativ ausgeglichen ist, wobei tendenziell mehr Aussagen auf der Seite der Ressourcen liegen. Hierzu zählen insbesondere die Cluster 6 und 2, die ausschließlich Ressourcen thematisieren. Cluster 5 und 1 setzen sich im Gegensatz dazu ausschließlich aus Arbeitsanforderungen zusammen. Zieht man die bewertete Wichtigkeit hinzu, so fällt zudem auf, dass die Cluster mit Ressourcen insgesamt überdurchschnittlich oft als wichtig bewertet wurden und die Cluster mit Arbeitsanforderungen dagegen die niedrigsten Werte auf der Bewertungsskala aufweisen. Wird daraufhin eine Prüfung signifikanter Unterschiede zwischen der Bewertung der vier Cluster über eine Varianzanalyse mit Welch-Korrektur vorgenommen, so unterscheiden sich Cluster 6 und 5 im Games-Howell-Test signifikant voneinander (0,47; 95 %-Konfidenzintervall [KI]: 0,19–0,75; *p* *<* 0,001). Zusammengenommen kann daher angenommen werden, dass die Teilnehmer*innen durchschnittlich ein eher positives Bild der digitalen Mediennutzung in der Schule in Bezug auf die Befindlichkeit von Lehrkräften haben. Dies ist insofern überraschend, als positive Auswirkungen der digitalen Mediennutzung auf Stress und Wohlbefinden wie zuvor dargestellt in der bisherigen Forschung keinen großen Stellenwert hatten. Diese Befunde können als Hinweis für die Wichtigkeit positiver Aspekte der digitalen Mediennutzung für die Entstehung von Stress und Wohlbefinden angesehen werden.

Bezogen auf die zweite Dimension entlang der y‑Achse kann festgestellt werden, dass ein Großteil der Aussagen Faktoren beschreibt, die auf der Ebene der einzelnen Lehrkraft verortet sind (Cluster 1, 2, 4–6, 7). Faktoren, die sich auf die Schulebene und die Ebene der Schüler*innen beziehen, wurden weitaus weniger häufig generiert (Cluster 3 bzw. Cluster 8 und teilweise Cluster 6). Verknüpft man diese dimensionale Anordnung wiederum mit der bewerteten Wichtigkeit, so fällt auf, dass Faktoren der Schulebene die im Durchschnitt höchste Wichtigkeit zugewiesen wurden (*M* = 3,12; *SD* = 0,40). Dieser Befund kann in Einklang mit aktuellen Forschungsbefunden gesehen werden, in denen angenommen wird, dass die schulischen Gegebenheiten eine wichtige Rolle für die Entstehung von digitalem Stress spielen [34].

Weiterhin war die Bewertung der Wichtigkeit einzelner Aussagen von Interesse. Aufgrund der hohen Anzahl an Aussagen werden im Folgenden lediglich diejenigen dargestellt, die vergleichsweise weit in Richtung des Skalenpols *wichtig* (Werte > 3) bzw. *unwichtig* (Werte < 2,25) liegen und unterschiedliche Faktoren adressieren. Aufgrund der linksschiefen Verteilung der Kennwerte (Schiefe: −0,28) wurden die Cut-off-Werte auf den Enden der Bewertungsskala nicht gleichmäßig gesetzt.

Durchschnittlich am wichtigsten bewertet wurden Aussagen zur technischen Ausstattung (A4: „Ausstattung mit digitalen Geräten an Schulen“) und zur digitalen Infrastruktur (A54: „WLAN-Empfang in der Schule“). Dies überrascht nicht, da das Vorhandensein von funktionierender Technik zweifelsfrei eine Grundvoraussetzung für eine erfolgreiche Anwendung digitaler Medien in der Schule ist. Ein weiterer Faktor mit einer im Durchschnitt hohen bewerteten Wichtigkeit bezieht sich auf eine erhöhte Arbeitsflexibilität (A10: „digitale Medien als Hilfsmittel bei Homeschooling oder in Krankheitsfällen“). Die Teilnehmenden sahen damit die positiven Aspekte flexiblerer Arbeitsmöglichkeiten als wichtiger an als potenzielle Gefahren, die damit einhergehen können. Zu nennen ist hier die Studie von Mauss [32], in der Lehrkräfte die Digitalisierung mit einer erschwerten Abgrenzung von Arbeit und Freizeit verbinden. Diese negative Seite der Arbeitsflexibilität kommt in dieser Studie nur in der Aussage „permanenter außerschulischer Austausch mit Schüler*innen per Mail könnte Lehrkraft stressen“ (A56) zum Tragen, deren durchschnittliche Wichtigkeit jedoch deskriptiv niedriger ausfiel. Weiterhin wurde der Wichtigkeit von Aussagen Ausdruck gegeben, die sich auf Vorteile für die Unterrichtsgestaltung (A18: „vielfältige Möglichkeiten der Unterrichtsgestaltung“), bessere Möglichkeiten der individuellen Förderung (A62: „bessere/mehr Möglichkeiten zur individuellen Förderung der Schüler*innen“) und einem für Schüler*innen attraktiveren Unterricht beziehen (A65: „Abwechslung zum regulären Unterricht macht Schüler*innen Spaß“). Damit wird angenommen, dass nicht nur Schüler*innen von einem digital unterstützten Unterricht profitieren, sondern sich dies auch positiv auf das Wohlbefinden von Lehrkräften auswirkt. Eine weitere Gruppe von Aussagen, die sich auf (fehlende) digitale Kompetenzen bezieht, ist durchschnittlich ebenfalls als wichtig angesehen worden (A53: „Wissen zum Umgang mit unterrichtsstörender Mediennutzung“). Dieser Befund erscheint wenig überraschend, da die digitalen Kompetenzen der Lehrkraft – ähnlich wie die angeführte digitale Ausstattung und Infrastruktur – als grundlegend für einen gelungenen Einsatz digitaler Medien gesehen werden können. Zudem liegt zu diesem Faktor eine vergleichsweise breite Befundlage aus bisheriger Forschungsliteratur vor. Aussagen zur Zeitersparnis (A27: „Materialaustausch über Online-Plattformen erspart Zeit“) und zur Vereinfachung von Arbeitsabläufen (A36: „Vorteil beim Aufbewahren und Speichern der Materialien“) wurden von den Teilnehmenden ebenfalls als wichtige Ressourcen wahrgenommen. Sowohl die Zeitersparnis als auch eine Arbeitserleichterung wurden in der Literatur bereits als Vorteile digitaler Medien ausgemacht [10, 32, 52]. Mit den Ergebnissen konnten nun erstmals Hinweise auf einen positiven Zusammenhang mit dem Wohlbefinden von Lehrkräften aufgezeigt werden. Zwar assoziierten Studien die Nutzung digitaler Medien auch mit Mehrarbeit und einer erhöhten Arbeitsbelastung [18, 31, 32]. Allerdings wurden entsprechende Aussagen (A67: „Umstellung des Unterrichts wird möglicherweise zu viel Arbeitsaufwand führen“) in dieser Studie deskriptiv als weniger wichtig bewertet. Schließlich wurde die Einstellung der Lehrkraft gegenüber digitalen Medien als wichtig eingestuft (A98). Dies erscheint insofern stimmig, da auch empirisch eine negative Einstellung mit digitalem Stress in Verbindung gebracht wurde [1, 45]. Eine positive Einstellung könnte als übergeordneter Faktor die Motivation unterstützten, sich mit digitalen Medien auseinanderzusetzen und sich digitale Kompetenzen anzueignen.

Eine Vielzahl an Aussagen wurde dagegen vom Neutralpunkt der bipolaren Bewertungsskala in Richtung des Pols *unwichtig* verortet. Am stärksten bestritten wurde die Wichtigkeit folgender Aussagen: „Wahrnehmung von nicht selbst initiierter Veränderung (dem Aufbruch ins digitale Zeitalter) als Bedrohung“ (A9); „Frust durch Wettbewerb zwischen Lehrkräften“ (A93); „Ressourceneinsparung (Papier, Kreide, …)“ (A37); „Gewohnheiten der Lehrkräfte“ (A11); „Angst der Lehrkraft, dass fließendes Schreiben auf Papier verlernt wird“ (A60); „Lehrkräfte müssen ihr Rollenverständnis weiterentwickeln“ (A14); „Stress und Unsicherheit aufgrund der Sorge um Datensicherheit“ (A8); „neue Lehrkräfte haben Schwierigkeiten, sich in digitale Organisationsformen einzufinden“ (A55) sowie „Einsatz von digitalen Medien erleichtert das tägliche Gepäck von Lehrkräften“ (A24). Da zuvor bereits angeführt wurde, dass Clustern mit Arbeitsanforderungen im Durchschnitt eine geringere Wichtigkeit zugeschrieben wurde, überrascht es nicht, dass die meisten dieser Faktoren Arbeitsanforderungen darstellen. Handelt es sich hingegen um Ressourcen, so fällt auf, dass diese sehr spezifische Faktoren adressieren und deren Wichtigkeit möglicherweise daher eher abgelehnt wurde.

Zusammenfassend lässt sich feststellen, dass eine Vielzahl an Faktoren generiert wurde, die bei der Nutzung digitaler Medien im schulischen Kontext von Lehrkräften relevant sein könnte. Es konnten erste empirische Hinweise für eine positive Wirkung von Vorteilen digitaler Medien für das Wohlbefinden von Lehrkräften aufgezeigt werden. Die Ergebnisse zur Arbeitsflexibilität, Zeitersparnis und Mehrarbeit durch digitale Medien stehen im Widerspruch zu vorhandenen Forschungsbefunden zu digitalem Stress. Diese weisen darauf hin, dass weitere Studien zu Einflussfaktoren vonnöten sind, um differenziertere Aussagen treffen zu können, unter welchen Bedingungen Lehrkräfte bestimmte Faktoren als Ressource oder als Arbeitsanforderung wahrnehmen.

## Limitationen

Bezüglich der rekrutierten Stichprobe ist darauf hinzuweisen, dass diese im Vergleich zu regulären Lehrkräften geringere praktische Erfahrungen besitzt. Während nicht anzunehmen ist, dass die Strukturierung der Aussagen dadurch tangiert wird, könnte die eingeschätzte Wichtigkeit der Aussagen aufgrund unterschiedlicher Erfahrungs- und Altershintergründe bei aktiven Lehrkräften anders ausfallen. Weiterhin ist zu berücksichtigen, dass eine große Mehrheit der Teilnehmenden angehende Lehrkräfte für die Grundschule war. Da digitale Medien in der Grundschule teilweise anders genutzt werden als in weiterführenden Schulen [21], könnte dies Auswirkungen auf die generierten Aussagen und die eingeschätzte Wichtigkeit der Aussagen gehabt haben. Aus diesen Gründen ist eine weitere Studie mit regulären Lehrkräften aus allgemeinbildenden Schulen in Planung.

## Fazit für die Praxis

- Es konnte eine breite Zusammenstellung und Strukturierung von Arbeitsanforderungen und Ressourcen der digitalen Mediennutzung im schulischen Kontext für Lehrkräfte in Deutschland erzeugt werden.
- Positive Folgen der digitalen Mediennutzung für Stress und Wohlbefinden von Lehrkräften wurden wichtiger eingeschätzt als negative Folgen. Hier scheinen weitere Untersuchungen zu einzelnen Einflussfaktoren vielversprechend.
- Die Studie unterstützt frühere Befunde zur Wichtigkeit der schulischen Rahmenbedingungen, insbesondere zur technischen Ausstattung und zu schulischen Unterstützungsmöglichkeiten.
- Widersprüchliche Befunde mit einzelnen früheren Forschungsergebnissen weisen darauf hin, dass weitere Studien zu Einflussfaktoren vonnöten sind, um differenziertere Aussagen zur Wirksamkeit einzelner Faktoren treffen zu können.

## Supplementary Information

Online-Material 1: Komplette Liste der generierten Aussagen

## Footnotes

1 Die komplette Liste der generierten Aussagen steht als Onlinematerial zur Verfügung (Onlinematerial 1).

2 Einzelne Aussagen werden durch den Buchstaben A (= Aussage) und einer Identifikationsnummer abgekürzt.
