# Supplementary material for: Job demands and resources of digital media use among teachers: A group concept mapping study with trainee teachers
Source: Pravent Gesundh. 2023 Feb 9:1–11. [Article in German] Online ahead of print. doi: 10.1007/s11553-023-01015-w (PMC9910262; doi:10.1007/s11553-023-01015-w)
Supplement: Supplementary file 1 — Online-Material 1: Komplette Liste der generierten Aussagen [file 11553_2023_1015_MOESM1_ESM.pdf]

Cluster 1: Persönliche Voraussetzungen und Herausforderungen im Umgang mit der Technik

ID	Aussage	Mean	SD	Cluster
A1	Lehrkraft muss immer auf dem neusten Stand sein und sich immer weiterbilden	3.08	0.77	1
A5	Differenz zwischen Kompetenzen der Lehrkräfte in der Mediennutzung	2.36	0.99	1
A9	Wahrnehmung von nicht selbst initiierter Veränderung (dem Aufbruch ins digitale Zeitalter) als Bedrohung	1.50	0.77	1
A12	keine entsprechende Vorbereitung für Lehrkräfte in Bezug auf digitale Medien und ihren Einsatz in der Schule	3.14	0.64	1
A23	ältere Lehrkräfte sind mit neuerer Technik meist überfordert	2.81	0.95	1
A44	Überforderungsgefühl durch geforderten Einsatz vieler neuer und eventuell für die Lehrperson unbekannter Medien	2.75	0.94	1
A50	Technikangst	2.28	1.14	1
A55	neue Lehrkräfte haben Schwierigkeiten, sich in digitale Organisationformen einzufinden	2.11	1.01	1
A85	Abhängigkeit von einem Gerät	3.00	0.93	1
A89	persönlicher Zweifel an Mehrwert der digitalen Medien im Schulalltag	2.14	0.68	1

Cluster 2: Erleichterung und Effizienz

ID	Aussage	Mean	SD	Cluster
A2	Erleichterung der Verwaltung/Organisation	2.78	0.76	2
A16	Erleichterung der Kommunikation mit Erziehungsberechtigten und Kolleg*innen	3.08	0.77	2
A24	Einsatz von digitalen Medien erleichtert das tägliche Gepäck der Lehrkräfte	2.14	0.99	2
A25	Zeitgewinn, da Lernsoftwares die Korrekturen übernehmen	2.53	0.94	2
A27	Materialaustausch über Online-Plattformen erspart Zeit	3.14	0.90	2
A29	Austausch mit Lehrkräften anderer Schulen wird erleichtert	2.92	0.94	2
A36	Vorteil beim Aufbewahren und Speichern der Materialien	3.25	0.84	2

A37	Ressourceneinsparung (Papier, Kreide, …)	1.92	0.84	2
A41	schneller Kontakt zu Schüler*innen außerhalb des Unterrichtes durch Nachrichtendienste möglich	2.36	0.93	2
A58	Entlastung in der Unterrichtsorganisation bei ausreichender Medienkompetenz	3.00	0.76	2
A64	platzsparend (Datenspeicher online)	2.50	1.00	2
A75	einfache, schnelle Datenübertragung (Cloud, externes Medium, ...)	3.08	0.77	2
A78	Lehrkräfte sparen Zeit, da sie Arbeitsblätter oder Elternbriefe direkt abändern können (ohne dauernd neue aufzusetzen)	2.92	1.02	2
A79	Lehrkräfte können Tafelbild zuhause vorbereiten, was Zeit in der Stunde spart	3.00	0.99	2
A91	digitale Medien als Entlastung für Lehrkräfte mit hohem Kompetenzgefühl	2.78	0.99	2
A97	durch Apps (z. B. schul.cloud) können Lehrkräfte der gleichen Schule sich austauschen und gegenseitig so leichter unterstützen	2.92	0.87	2

Cluster 3: Technische Ausstattung und Rahmenbedingungen für einen gelingenden Einsatz digitaler Medien

ID	Aussage	Mean	SD	Cluster
A3	Bildungsserver, auf dem Unterrichtsmaterial zur Verfügung steht	2.75	0.97	3
A4	Ausstattung mit digitalen Geräten an Schulen	3.69	0.67	3
A7	Hilfsangebote im Kollegium	2.89	0.85	3
A15	Werden Programme vorgegeben oder hat man die freie Wahl?	2.36	1.02	3
A19	Offenheit der Schulleitung bei Vorschlägen zur digitalen Lehre	2.94	1.01	3
A20	Programme zur Unterrichtsgestaltung werden von der Schule zur Verfügung gestellt, z. B. Aufgabenblatt selbst designen	2.72	0.91	3
A26	gute schulische (digitale) Infrastruktur	3.47	0.88	3
A31	Technische Ressourcen der Lehrkraft zuhause	3.08	1.08	3
A35	zeitgemäße Technik, z. B. keine veralteten Betriebssysteme	3.31	0.89	3
A48	Transparenz im Funktionieren/Aufbau	2.53	0.94	3
A54	W-LAN Empfang in der Schule	3.64	0.72	3

A59	ausreichende Organisation und Absprachen	2.86	0.90	3
A82	gute Einführung in die Verwendung digitaler Medien (bspw. bereits im Studium)	3.36	0.83	3
A90	ausreichend Endgeräte für Schüler*innen zur Verfügung	3.67	0.63	3
A94	Möglichkeit der Inanspruchnahme eines technischen Supports für Schulen bei Problemen/Fragen	3.19	0.86	3
A95	persönliche Weiterbildung für den Umgang mit Medien	3.06	0.63	3
A96	Angebot schulischer Fortbildungen	3.44	0.77	3

Cluster 4: Selbstwahrnehmung und Engagement der Lehrkraft in Bezug auf den Einsatz digitaler Medien im Unterricht

ID	Aussage	Mean	SD	Cluster
A6	Motivation, neue Dinge auszuprobieren	2.61	0.87	4
A13	ob digitale Medien wirklich zugunsten individuellen Lernens genutzt werden, oder lediglich als alternative Darstellungsform zu "klassischen" Unterrichtsmedien	2.31	0.71	4
A34	Selbstsicherheit	2.56	1.03	4
A45	Offenheit für neue Arbeitstechniken	2.89	0.92	4
A46	hohe Kompetenzwahrnehmung fördert Bereitschaft der Auseinandersetzung mit digitalen Medien	2.61	0.87	4
A76	Erweiterung des eigenen Horizonts	2.47	0.74	4

Cluster 5: Psychische und physische Belastungen sowie Risiken in Folge des Einsatzes digitaler Medien

ID	Aussage	Mean	SD	Cluster
A8	Stress und Unsicherheit aufgrund der Sorge um Datensicherheit	2.08	0.84	5
A17	Angst bezüglich digitaler Medien etwas Falsches zu vermitteln oder etwas schlecht zu vermitteln	2.31	0.98	5
A32	mögliche Reduktion der direkten Interaktion mit Klassen kann zu Frust führen	2.36	0.87	5

A33	Möglichkeit der Schüler*innen, etwaige Fehltritte (z. B.: falsches Verhalten, fachliche Fehler, …) der Lehrkraft aufzuzeichnen	2.31	0.98	5
A43	Cybermobbing gegen die Lehrkraft wird möglich	2.86	1.13	5
A47	digitale Ungleichheit der Schüler*innen als Belastung für die Lehrkraft	2.78	0.96	5
A49	eigenständiges Einarbeiten in neue digitale Medien kostet Zeit und Nerven	2.94	0.95	5
A51	individueller Umgang mit den Tablets der Schüler*innen stressig für Lehrkraft, wenn jede*r Schüler*in etwas anderes macht	2.61	0.93	5
A56	permanenter außerschulischer Austausch mit Schüler*innen per Mail könnte Lehrkraft stressen	2.67	0.89	5
A60	Angst der Lehrkraft, dass fließendes Schreiben auf Papier verlernt wird	2.03	1.03	5
A67	Umstellung des Unterrichts wird möglicherweise zu viel Arbeitsaufwand führen	2.64	0.99	5
A68	Kontrollverlust (indem man nicht sehen kann, was die Schüler*innen am Tablet o. Ä. machen)	2.75	0.94	5
A69	Überforderung, wenn Schüler*innen mehr Kompetenzen mit technischem Gerät haben als Lehrkraft	2.47	0.91	5
A72	Druck aufgrund der Erwartungen der Gesellschaft im Umgang mit neuen/digitalen Medien	2.28	0.81	5
A73	Stress durch unterschiedliche Bedürfnisse der Schüler*innen aufgrund deren Vorkenntnisse im Umgang mit Medien	2.39	0.64	5
A77	Müdigkeit/Schlappheit durch hohe Bildschirmzeit	2.94	0.89	5
A84	Augenschmerzen und Kopfschmerzen durch hohe Bildschirmzeit	2.94	0.92	5
A86	Stress und Druck sich selbst informieren zu müssen	2.53	0.77	5
A93	Frust durch Wettbewerb zwischen Lehrkräften	1.67	0.89	5

Cluster 6: Vielfalt, Flexibilität und Effizienz bei der Gestaltung des Lehr-Lern-Prozesses

ID	Aussage	Mean	SD	Cluster
A10	digitale Medien als Hilfsmittel bei Homeschooling oder in Krankheitsfällen	3.47	0.74	6
A18	vielfältige Möglichkeiten der Unterrichtsgestaltung	3.36	0.90	6
A21	Einsatz von digitalen Medien erleichtert die Recherche	3.03	0.97	6
A22	digitale Medien ermöglichen effizienteres Arbeiten im Unterricht	2.89	0.95	6
A28	gute Möglichkeiten, die digitalen Medien in den Unterricht zu integrieren	2.83	0.85	6
A30	Schülerfreundlichkeit	3.08	1.00	6
A39	visuelle und auditive Darstellung möglich	3.06	0.92	6
A42	Videos in Bibliothek für Lehrerkräfte enthalten, um z. B. in Chemie gefährlichere Experimente trotzdem zeigen zu können	2.64	0.87	6
A52	Gewinnung der Aufmerksamkeit der Schüler*innen	2.53	0.81	6
A61	zeitgemäßer Unterricht	2.75	0.91	6
A62	bessere/mehr Möglichkeiten zur individuellen Förderung der Schüler*innen	3.19	0.89	6
A63	mehr Kontrollgewinn über den Lernerfolg und -prozess durch Schaffung weiterer Strukturen (Lernplattformen, Software, Online-Wettbewerbe) für die Schüler*innen zur Nutzung von Lerninhalten	3.00	0.79	6
A65	Abwechslung zum regulären Unterricht macht Schüler*innen Spaß	3.14	0.54	6
A71	fordert selbstgesteuertes Lernen	2.83	0.91	6
A83	bessere Möglichkeiten der aktiven Teilnahme innerhalb der passiven Unterrichtsphasen	2.81	0.75	6
A87	Ermöglichung räumlicher Mobilität und Flexibilität	2.97	0.88	6
A88	Informationen können für Schüler*innen zugänglicher aufbereitet werden	3.00	0.86	6
A99	Methodenvielfalt	3.06	0.86	6

Cluster 7: Individuelle Kompetenzen und Anpassungsbereitschaft der Lehrkraft

ID	Aussage	Mean	SD	Cluster
A11	Gewohnheiten der Lehrkräfte	1.97	0.94	7
A14	Lehrkräfte müssen ihr Rollenverständnis weiterentwickeln	2.08	1.13	7
A38	fehlende Medienkompetenz	3.28	0.85	7
A40	Wissen von Anwendungsmöglichkeiten	3.19	0.89	7
A53	Wissen zum Umgang mit unterrichtsstörender Mediennutzung	3.31	0.92	7
A57	Möchte die Lehrkraft wirklich mehr digital arbeiten (oder macht sie das nur, weil es so gewünscht wird)?	2.47	1.08	7
A70	ob im privaten Gebrauch viele digitale Medien genutzt werden oder nicht	2.47	1.08	7
A80	individuelle Kenntnisse im Umgang mit digitalen Medien	3.03	0.88	7
A81	Zufriedenheit mit traditionellem Unterricht	2.25	0.87	7
A92	allgemeines Interesse am Umgang mit digitalen Medien	2.69	0.86	7
A98	generelle Einstellung gegenüber digitalen Medien	3.08	0.94	7

Cluster 8: Individuelle Merkmale der SuS

ID	Aussage	Mean	SD	Cluster
A66	computerbezogene Kompetenzen von Schüler*innen	2.83	0.94	8
A74	Alter der Schüler*innen	2.50	1.53	8
